# Biosensing Strategies to Monitor Contaminants and Additives on Fish, Meat, Poultry, and Related Products

**DOI:** 10.3390/bios15070415

**Published:** 2025-06-30

**Authors:** Zenebe Tadesse Tsegay, Elahesadat Hosseini, Teresa D’Amore, Slim Smaoui, Theodoros Varzakas

**Affiliations:** 1 Department of Food Science and Post-Harvest Technology, College of Dryland Agriculture and Natural Resources, Mekelle University, Mekelle P.O. Box 231, Ethiopia; zenebe.tadesse@mu.edu.et; 2 Department of Chemical Engineering, Payame Noor University, Tehran 19395-4697, Iran; hosseinielahe14@gmail.com; 3 Laboratory of Preclinical and Translational Research, IRCCS CROB, Centro di Riferimento Oncologico della Basilicata, 85028 Rionero in Vulture, Italy; 4 Laboratory of Microbial and Enzymes Biotechnology and Biomolecules (LMEBB), Centre of Biotechnology of Sfax (CBS), University of Sfax-Tunisia, Road of Sidi Mansour Km 6, P.O. Box 1177, Sfax 3018, Tunisia; slim.smaoui@cbs.rnrt.tn; 5 Department of Food Science and Technology, University of the Peloponnese, Antikalamos, 24100 Kalamata, Greece

**Keywords:** biosensor, fish, meat, quality, monitoring, standardization

## Abstract

Biosensors have emerged as highly sensitive, rapid, and specific tools for detecting food safety hazards, particularly in perishable products, such as fish, meat, and poultry. These products are susceptible to microbial contamination and often contain additives intended to improve shelf life and flavor, which may pose health risks to consumers. Recent advances in biosensor technologies integrated with smartphones, artificial sensing systems, 3D printing, and the Internet of Things (IoT) offer promising solutions for real-time monitoring. This review explores the types, mechanisms, standardization approaches, and validation processes of biosensors used to detect contaminants and additives in animal-based food products. Furthermore, the paper highlights current challenges, technical limitations, and future perspectives regarding the broader implementation of biosensors in modern food safety monitoring systems.

## 1. Introduction

**Figure 1:**
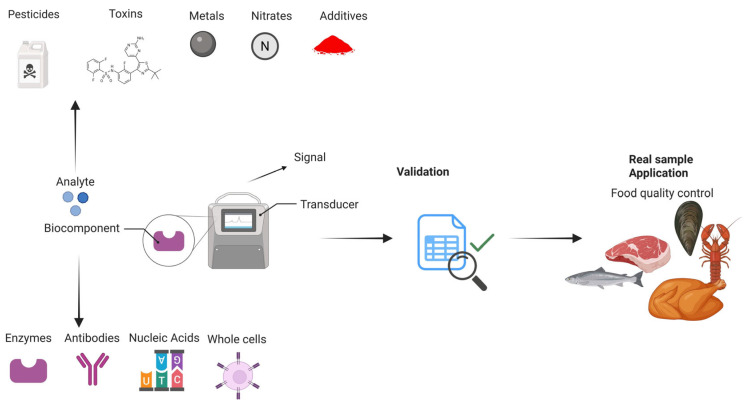
Schematic representation of meat and fish sample, target analytes, biorecognition elements, and an analytical method.

A wide range of contaminants, including physical, allergenic, environmental, chemical, and biological agents, can be found in fish, meat, poultry, and related products. According to the World Health Organization (WHO), approximately 600 million people fall ill and 420,000 die each year due to foodborne diseases [1]. The consumption of fish, meat, poultry, and related products with compromised quality and safety can significantly increase the risk of foodborne illnesses. Numerous chemical and biological contaminants have been reported in such products, including antibiotic residues, toxic chemicals, food additives, heavy metals, pathogens, and pesticides. For instance, nitrate has been detected in meat samples [2]; chloramphenicol, an antibiotic residue, has been found in beef and pork samples [3]; and pathogens, such as *Salmonella enterica*, *Listeria monocytogenes*, and *Escherichia coli,* have been identified in ready-to-eat beef, chicken, and turkey breast meat [4]. Other reported issues include the adulteration of donkey meat in cooked sausages [5], and the presence of spoilage biogenic amines, such as hypoxanthine (Hx), in fish samples [6]. Rapid and accurate detection of food contaminants, as an alternative to traditional culture-based techniques, is gaining increasing emphasis on current food quality and safety management systems. Conventional methods for detecting food contaminants, such as culture-based techniques, antibody-based immunoassays, fatty acid and protein profiling, chromatographic separations, and spectroscopic analyses, face several limitations. These include labor-intensiveness, high costs associated with chemical reagents, time-consuming procedures, the need for trained personnel, and limited applicability for on-site detection due to their dependence on laboratory infrastructure [1]. Figure 1 illustrates the target analytes in meat and fish samples, along with the types of bioreceptors and the corresponding measurement techniques. Biosensors are analytic devices employed to analyze, record, and transform biochemical information by controlling the interaction of immobilized bioreceptors and chemical components from pathogenic or naturally produced or additives used in foods [7,8]. Applications of food biosensors in intelligent packaging—such as for labeling, microbial spoilage detection, time–temperature indicators, nanosensors, and barcodes—are becoming increasingly common at industrial and commercial levels [9]. Biosensors are classified based on their measurement principles and the types of transducers used for real-time monitoring. These include: physical biosensors (which detect changes in mass, pressure, strain, or force), electrical biosensors (which measure variations in electrical distribution), calorimetric biosensors (which monitor changes in heat), optical biosensors (which detect changes in light), magnetic biosensors (which respond to changes in magnetic fields), and ion channel switch biosensors (which detect functional molecular interactions) [1]. Another classification of biosensors is based on the type of biorecognition element used to detect target analytes in the quality and safety monitoring of fish, meat, and poultry-related products. Enzyme-based biosensors, immunosensors, and DNA-based biosensors are among the most common types in this category, as described by Nami et al. [7]. Biosensors can also be classified based on their readout mechanisms, which include acoustic wave sensors, surface plasmon resonance (SPR), and mass spectrometry, as well as label-based approaches, such as fluorescence and chemiluminescence, as designated by Nanda et al. [9].

Standardization and validation of biosensors in fish, meat, poultry, and related product quality and safety monitoring for better reliability, reproducibility, and regulatory acceptance are mandatory at commercial and industrial levels. The complexity of food matrices and interference of environmental and biochemical changes could reduce the reliability and reproducibility of biosensors. Hence, calibration and standardization are regular approaches that should be established. For instance, heat-transfer biosensors employed to detect trace levels of chemical additives in dairy were calibrated to consistent sensitivity and reproducibility [10]. Although various types of biosensors have been developed to monitor fish and meat samples, their integration into regulatory and commercial systems, like HACCP, ISO 22000:2018 [11], and Codex Alimentarius, has not yet been fully realized due to limitations in standardization and validation. This review is intended to share insights on the biosensing strategies of biosensors for monitoring contaminants and additives in fish, meat, poultry, and related products. Moreover, a brief discussion on standardization and validation of biosensors in real-time quality analysis as well as current challenges, technical limitations, and future perspectives of biosensor utilization have been addressed.

## 2. Biosensors and Monitoring Strategies of Fish, Meat, Poultry, and Related Product Quality Parameters

Biosensors contain integral parts, such as a biorecognition element, transducer/electrode, and data visualization device. The biorecognition elements are suitable for detecting target analytes. To date, different types of biosensors that are applicable for monitoring pesticides/drug residuals, toxins, heavy metals, nitrates, additives (adulterations) in meat and fish samples have been developed [12]. As depicted in Figure 1, enzyme-, antibody-, nucleic acid-, and whole cell-based biorecognition elements are common for developing biosensors. Understanding the electrochemistry of biosensors in meat and fish quality monitoring is crucial for rapid and sensitive analysis. The analytical performance of biosensors depends on the chemical reactions of biorecognition elements and the target analyte, how it transduces measurable electrical signals, and the strategies applied for signal amplification [12].

### 2.1. Biosensor Development Strategies and Mechanism of Sensing

Biosensors are analytic devices employed to analyze, record, and transform biochemical information by controlling the interaction of immobilized bioreceptors and chemical components from pathogenic or natural products or additives used in foods [7].

Voltammetry, amperometry, potentiometry, spectroscopy, and impedance are some well-known electrochemical strategies for meat and fish quality monitoring [12]. The amperometric method works by measuring the constant potential due to the current generated by the redox reaction. For instance, the organophosphate detection using acetylcholinesterase in milk samples is through the inhibition of enzyme activity, which is directly proportional to the analyte concentration. The redox reaction between acetylcholinesterase and pesticide concentration generate a measurable decrease in the current [13]. The potentiometric method is an uncomplicated electrochemical measurement. It measures a potential difference in response to the ion concentration generated between a working and reference electrode employing a selective membrane. This method is valid for monitoring a broad range of ions, like lead, mercury, and cadmium, in meat samples [14]. Electrochemical impedance spectroscopy (EIS) is a common spectroscopic technique employed for detecting label-free biohazards and chemical contaminants in poultry products. It works by measuring the impedance change that is generated due to the selectively binding effect of the target pathogen on the electrode surface of the immobilized antibodies. For instance, this method was applied to detect *Salmonella* in poultry products [15]. Voltammetry is an electrochemical technique in which the current generated during an electrochemical reaction is measured as a function of the applied voltage to a working electrode. This current, which can be cathodic (due to reduction) or anodic (due to oxidation), provides information about the electrochemical process and the analyte being studied. For example, the voltametric technique shows significant sensitivity and specificity to measure antibiotics, like pefloxacin, in shrimp and pork samples. This technique has been applicable for developing novel portable electrochemical sensors to monitor pefloxacin in food samples [16].

**Figure 2:**
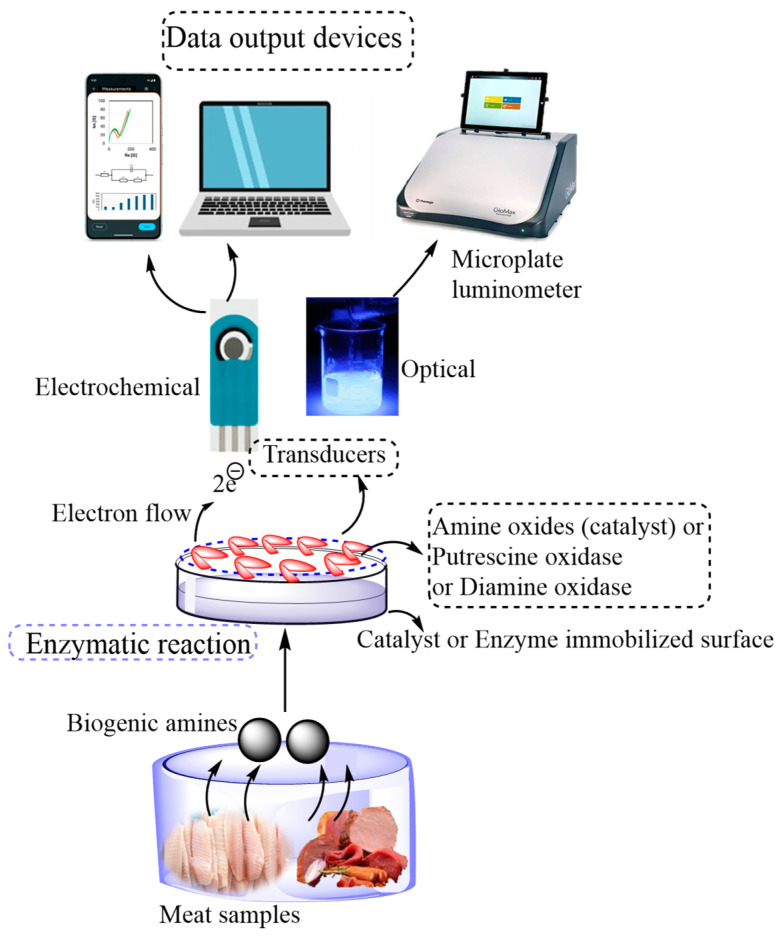
Diagram illustrating the detection of biogenic amines in meat samples.

**Figure 3:**
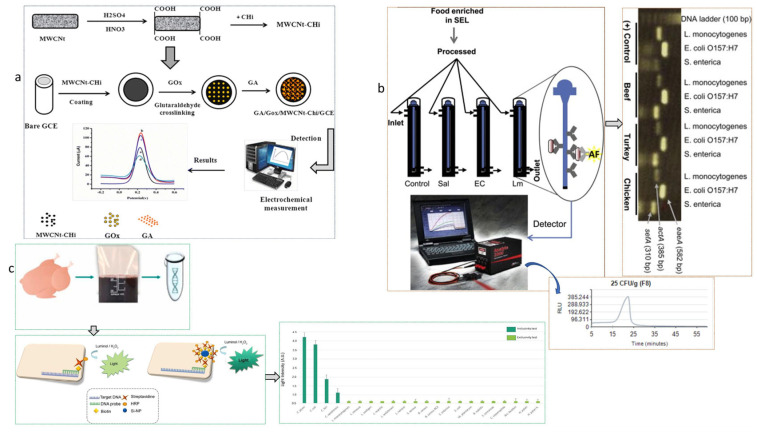
Typical biosensors and their mechanism of sensing. (**a**) Illustration of the stepwise glucose biosensor preparation process and electrochemical measurement of glucose reduction in fresh meat (GCE = glassy carbon electrode; MWCNt = multi-walled carbon nanotubes; CHi = chitosan; BSA = bovine serum albumin; GOx = Glucose oxidase; GA = glutaraldehyde) (reproduced from Uwimbabazi et al. [22] with permission from the Journal of Food Analytical Methods, copyright 2017). (**b**) Multi-pathogen detection strategy in meat samples using a fiber optic sensor with PCR confirmation (Sal = *Salmonella*; EC = *E. coli*; Lm = L. *monocytogenes*; AF = Afucosylation) (reproduced from Ohk and Bhunia [4] with permission from Food Microbiology, copyright 2013). (**c**) Paper-based DNA biosensor for *Campylobacter* detection using a biotinylated probe, streptavidin-HRP dot blot read-out, and functionalized biotin-Si-NPs amplification (biotin-Si-NPs = biotinylated silica-nanoparticles; HRP = horseradish peroxidase) (reproduced from Vizzini et al. [23] with permission from Biosensors and Bioelectronics, copyright 2021).

Bioreceptors are incorporated into biosensors as reversible and irreversible immobilization strategies [1]. The presence of chemical components, such as xanthine, histamine in fish and fishery products, and pathogens, like *Salmonella* species in poultry products and *Echerichia* coli (*E. coli*) in ground beef, are the main sources of food-borne diseases [7]. Hence, monitoring their availability and standardizing their permissible limits are very crucial. Bioreceptors developed by reversible immobilization employing proteins and enzymes are applicable for generating biorecognition elements. These biorecognition elements easily detach from the sensing surface to be linking and binding agents during the reuse of biosensors [1,17]. However, the biorecognition element (bioreceptors) made by irreversible immobilization has strong crosslinking, entrapment, and covalent bonding mechanisms [17,18]. Irreversible immobilization of bioreceptors helps develop highly stable biorecognition elements, although they have significant limitations, such as loss of enzyme activity, toxicity of linkers used, and demand for high purity enzymes [1]. Figure 2 shows the detection of biogenic amines in meat samples with different measurement techniques. Moreover, Figure 3 illustrates the strategies of the glucose biosensor preparation process and electrochemical measurement of glucose reduction; multi-pathogen detection strategy using a fiber optic sensor; and paper-based DNA biosensor for *Campylobacter* detection in meat and fish samples.

Biosensors contain a bioreceptor and a transducer as two major parts for the accurate detection of chemical components and transform biochemical information into electrical or optical signals. These bioreceptors are immobilized with nucleic acids, antigens, hormones, or enzymes, molecularly imprinted polymers (MIP), or chemoresponsive dyes, such as chemical/natural pH dyes, conjugated polymers, colorimetric sensor arrays, and fluorophores for recognizing and identifying each target element. In contrast, the transducer helps to transform the biochemical information into electrical or optical signals later measured employing colorimetric or electroanalytical devices [7]. The sensing mechanism of these biosensors are based on the reaction of active sites with the bioreceptor (immobilized biorecognition) as biological or organic material) and the substrate from the tested food material. The electrons produced due to the chemical reaction create a medium of electron flow on the surface of the electrode so that the transducer transforms them as response signals. A typical sensing mechanism of biogenic amines for monitoring the quality and safety of meat is presented by Nami et al. [7]. First, biogenic amines present in a meat product are oxidized into hydrogen peroxide (H_2_O_2_), NH _3_, and aldehyde in the presence of oxygen and water using amine oxides as a catalyst (Equation (1)). Next, by applying a high potential, the produced H_2_O_2_ is dissociated into 2 hydrogen ions and oxygen creating 2 electrons. Then, the generated 2 electrons are used as electron flow, providing response signals by the surface of the electrode.



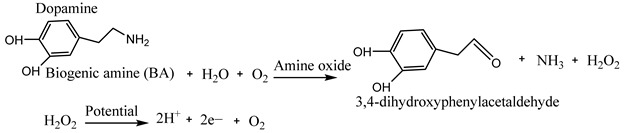

(1)


A similar sensing mechanism for monitoring histamine in fish spoilage is presented in Equation (2) [19]. First, histamine is oxidized into imidazole acetaldehyde, NH_3_, and H_2_O_2_ using diamine oxidase (DAO). Then, the produced H_2_O_2_ is dissociated into 2 hydrogen ions and oxygen and creates 2 electrons, which are used as electron flow, providing response signals by the surface of the electrode.



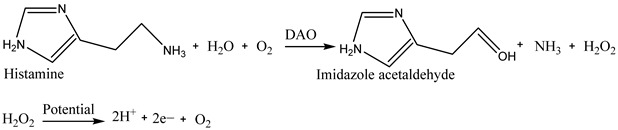

(2)


Omanovic-Miklicanin and Valzacchi [20] developed chemiluminescence biosensors to detect the presence of putrescine (Put) and cadaverine in beef, pork, chicken, turkey and fish meat samples. They used putrescine oxidase or diamine oxidase as biorecognition elements (bioreceptors) and a microplate luminometer as a detection device. The enzymatic reaction and biosensing mechanism are explained in Equation (3). Since the putrescine does not show chemiluminescence characteristics, its concentration in meat samples cannot be determined directly during the chemiluminescence reaction. However, it can be measured indirectly by measuring H_2_O_2_. Hence, first, putrescine is oxidized into H_2_O_2_, 4-aminobutanal, and NH_3_ in the presence of oxygen and water and using putrescine oxidase or diamine oxidase enzymes (Equation (3)). Then, H_2_O_2_ is reacted with luminol in an alkaline solution and using cobalt (II) chloride hexahydrate as a chemiluminescence catalyst. At last, this catalyzed chemiluminescence reaction of luminol with H_2_O_2_ creates 3-aminophthalate with light, and the created light is measured by employing a microplate luminometer (Equation (3)). The produced light intensity due to this reaction is proportional to the concentration of hydrogen peroxide.

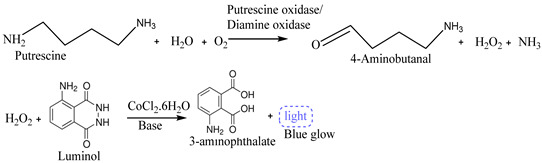
(3)

The presence of xanthine in chicken meat can be detected using an optical biosensor developed with guanine deaminase and xanthine oxidase (XOD) as biorecognition elements [21]. Briefly, XOD and dye phenol red indicator were co-immobilized into sol-gel-based circular plastic discs to develop the biosensor. The mechanism of this biosensing process involves the enzymatic oxidation of xanthine into uric acid and H_2_O_2_ in the presence of water, oxygen, and XOD as the catalytic enzyme (Equation (4)). The produced uric acid lowers the pH of the medium from approximately 7.5 to 6.0. This pH change can be visualized using phenol red as an absorptive dye, resulting in a noticeable color change.

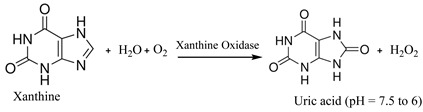
(4)

### 2.2. Types of Biosensors Employed to Monitor Fish, Meat, Poultry, and Related Product Quality Parameters

Several biosensors have been developed and introduced to the current world. These biosensors have different applications based on the target analyte intended to be determined. These have been employed for fish, meat, poultry, and related product quality and safety monitoring. Nami et al. [7] classified biosensors based on the type of bioreceptor (biorecognition element) utilized in enzyme-based biosensors, immunosensors, and DNA-based biosensors. Enzyme-based biosensors employ enzymes to create contact with sample analytes and produce a signal for measurement. Immunosensors use antibodies as a bioreceptor that are applicable for the detection of pathogens or toxins in meat samples. These antibodies contact the pathogen or toxin and create a signal for measurement. However, DNA-based biosensors are applicable for detecting DNA in meat samples using DNA as a biorecognition element. Here, the bioreceptor DNA interacts with the target DNA in the analyte. Some of the enzyme-based biosensors, immunosensors, DNA-based biosensors, and the bioreceptors employed for monitoring fish, meat, poultry, and related product quality and safety are summarized in Table 1.

Nanda et al. [9], on the other hand, classified biosensors based on the immobilization of biorecognition elements, types of transducers used, and detection techniques applied as label free and label based. Bioreceptor-based biosensors are developed by immobilizing enzymes, aptamers, whole cells, nanosensors, immunosensors, and antibodies. Electrochemical, optical, and mass-based/gravimetric biosensors are based on their transducers and used during real-time analysis of target analytes. Some transducers and electrodes employed with biosensors for fish and meat quality monitoring are presented in Table 1. On the other hand, label-free, such as acoustic wave, SPR, and mass spectrometry, and label-based bioreceptors, like fluorescence and chemiluminescence, are grouped based on their detection techniques.

Emerging biosensor technologies, such as smartphones, artificial sensing, 3D printing, and Internet of Things (IOT), are being applied as detection techniques for fish, meat, poultry, and related product quality and safety monitoring. Machine learning models are applicable in optical sensor-based methods considering color changes and water loss to predict beef quality [24].

## 3. Applications of Biosensors in Real-Time Food Quality Monitoring in Fish, Meat, and Meat Products

### 3.1. Biosensor-Based Detection of Freshness Indicators in Fish, Meat, and Meat Products

**Figure 4:**
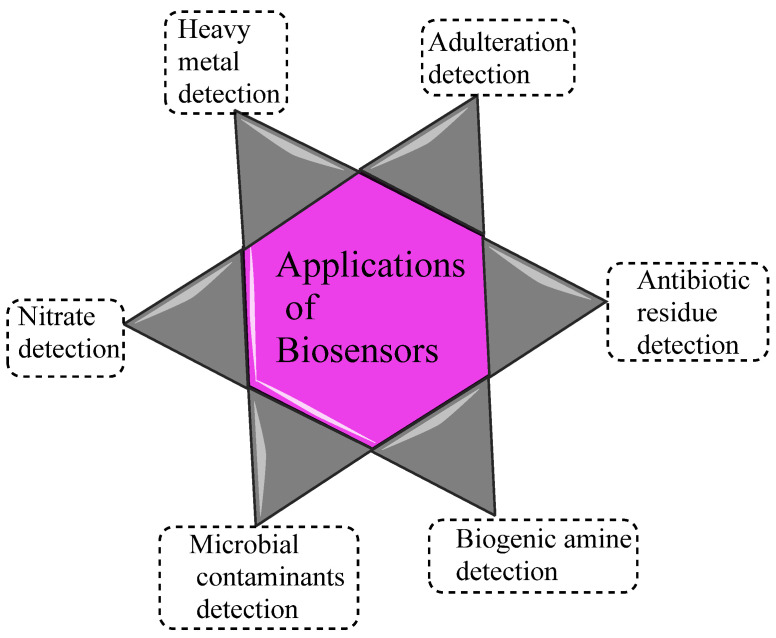
Applications of biosensors in meat and fish samples.

To measure the qualitative and quantitative characteristics of meat and meat product freshness, the visual appearance, pH, and meaty aroma are the major ones. In this line, metabolites, synthesized by chemical oxidation and microbial proliferation, could modify the quality of muscle food freshness. During storage, metabolites generated by microbial growth and chemical oxidation alter the quality and freshness of muscle foods [25,26,27,28]. Figure 4 shows existing applications of biosensors to monitor the freshness and quality of fish, meat, and meat products.

#### 3.1.1. Hypoxanthine

In the fish/meat industry, xanthine is exploited as a freshness indicator because of its accumulation in tissues after death. In order to monitor the freshness of pork, a TiO_2_ and graphene composite has been established [6]. The biosensor assesses the oxidation activity of XOD and Hx for seven days under refrigerated conditions [6]. Hx existence provokes a sour taste that is facile for detecting fish and meat sample degradation. Pierini et al. [29] developed an electroanalytical tool (edge plane pyrolytic graphite electrode) to determine the Hx, xanthine, and UA content in Argentinian fish samples. Similarly, to control the Hx content for pork meat freshness at several post-mortem periods, Guo et al. [30] developed an enzyme sensor by joining the O_2_ electrode and XOD. These authors reported that the produced biosensor displayed heightened sensitivity to Hx compared to HPLC analysis. By grafting reduced expanded graphene oxide (REGO) with Fe_3_O_4_ nanoparticles, Dervisevic et al. [31] produced a new amperometric xanthine biosensor and applied it to control fish freshness for 20 days. The xanthine concentration was detected at a range between 2 and 36 mM, at 3 s at detection limit equal to 0.17 mA/M. Interestingly, in 25-day-old fish samples, biosensors monitored 70% of its activity. In order to assess fish freshness with color marking by the unaided eye, XOD was employed in enzyme-mediated AuNR oxidation [32]. In this study, the color of the sensing system has a good link with the Hx level at a range between 0.05 and 0.63 mM. Chen et al. [33] proposed a fluorescence sensor derived from platinum nanoparticles (Pt NPs) to perceive Hx in aquatic products. At a [Hx] range between 8 and 2500 μM, the new biomaterial possesses a linear connection and detection limit of 2.88 μM. In meat samples, a μPAD biosensor was developed to detect Hx. The detection and quantitative limits were registered at 1.8 and 6.1 mg/L, respectively. The proposed assay exhibited a linear dynamic in the range of 5–40 mg/L. The analysis time was 5 min for triplicate measurement [34]. To assess meat freshness by Hx detection, Görgülü et al. [35] fabricated multi-enzyme biosensors. In this study, polypyrrole–polyvinyl sulphonate (PPy–PTS) films were synthesized on the platinum electrode surface by electropolymerization. The indicator enzymes, XOD and uricase, were immobilized within the polymer matrix. The registered amperometric response, at a potential of +400 mV, was attributed to the current resulting from the enzymatic oxidation of H_2_O_2_. The established biosensor displayed a minimum detection limit of 2.5 µM and a concentration range with a linear response of 2.5 to 10 µM. After 33 days of storage, the biosensor maintained 65% of its initial performance, demonstrating acceptable long-term stability for practical applications. This indicates the reusability and long-term stability of the developed enzyme electrode, considering storage conditions. For the evaluation of Hx in beef, chicken, fish, and pork meat, Devi et al. [36] evolved a biosensor designed with Au/Fe nanoparticles, and XOD was covalently grafted onto the electrode surface. At an optimal response within 3 s at pH 7.2 and 30 °C, the biosensor showed linearity in the range of 0.05 µM to 150 µM for Hx, with a detection limit of 0.05 µM. Using an absorption transmission approach, Garg and Verma [21] developed an optical biosensor for the detection of Hx. The assay is based on an enzymatic reaction catalyzed by XO, which converts xanthine into uric acid and H_2_O_2_. The formation of uric acid leads to a pH decrease, typically from 7.5 to 6.0. The researchers evaluated the xanthine content in chicken meat over a five-day storage period. As expected, xanthine levels increased progressively, indicating meat quality deterioration—from approximately 5 µM on day 1 to 44 µM on day 5. The method’s reliability was established in spiked samples, displaying a recovery between 94.2 and 96.5%. Zhang et al. [37] developed a new tool named electrochemiluminescence (ECL) of CdS quantum dots (QDs), combining electrochemistry and chemiluminescence. This technique indicated that electrical energy was used to launch a chemical reaction that generates light. These authors assembled and synthesized the new material onto poly (diallyldimethylammonium chloride)-functionalized carbon nanospheres (PFCNSs), leading to an increase in ECL intensity by dissolved O_2_ as a co-reactant. The sensor established a fast response with a linear range from 2.5 × 10^−8^ to 1.4 × 10^−5^ M and a detection limit of 5 nM (S/N = 3), and the obtained findings from fish sample analysis were closely matched to those from standard amperometric methods. The response time is the time it takes for a biosensor to respond to a change in the analyte concentration. The difference in sensing/analyzing time of the biosensors in the above discussion could be due to the type of biosensor, the analyte, and the transducer technology, which can influence the response time.

#### 3.1.2. Biogenic Amines and Volatile Amines

Biogenic amines, small organic compounds comprising one or more amino groups, are categorized into aliphatic, aromatic, and heterocyclic amines. These amines are mainly synthesized by the enzymatic decarboxylation of free amino acids or by amination and transamination of aldehydes and ketones [38].

The most prevalent biogenic amines present in aquatic and meat products are tyramine, cadaverine, putrescine, histamine, and trimethylamine [39]. Throughout muscle food deterioration, the formation of histamine, putrescine, and cadaverine are generally used as freshness indicators and can be monitored. Zhai et al. [40] created an amine-responsive bilayer film by using agar (AG), anthocyanins (AN), gellan gum (GG), and TiO_2_ nanoparticles for visual monitoring of meat spoilage. The AG-AN layer served as the detecting layer for volatile amines, while the AG-AN/GG-2%TiO_2_ film noticed trimethylamine (TMA) at a limit of 0.018 mM, a typical gas from meat spoilage. During the spoilage of silver carp and pork, the film exhibited a color variation ranging from rose-red to green, emphasizing its potential use in intelligent food packaging.

Based on the peroxidase-like activity of (Fe and Co) co-doped-CDs, Li et al. [41] developed a colorimetric tool to detect cadaverine and putrescine. With the enzymatic hydrolysis by diamine oxidase, biogenic amines were disintegrated to generate H_2_O_2_, which reacts with tetramethyl-benzidine with the catalysis of (Fe,Co)-co-doped CDs. The colorimetric method was used to perceive cadaverine and putrescine in various fish samples with a limit of 0.06 mg/kg. Checked by HPLC, the recoveries of the colorimetric method were confirmed by standards, signifying that the established colorimetric method was sensitive and accurate.

In order to monitor biogenic amines (BAs), Luo et al. [42] developed a hydrogel composed of β-d-glucose pentaacetate (β-D-GP), silver ions, and agarose. Under alkaline conditions, in contact with BAs, β-D-GP could be hydrolyzed to form β-d-glucose, which decreases silver ions to silver nanoparticles, and generates visible color variations. These changes can be analyzed with the naked eye or quantified using smartphone-based RGB (red/green/blue) analysis of fish samples. Polyaniline (PANI) synthesized via in situ chemical oxidative polymerization was spray-coated onto flexible interdigitated electrodes (IDEs) for observing ammonia gas and have been employed for checking food quality. The sensor’s electrical response increased linearly with increasing ammonia concentrations. It confirmed a constant linear response in the 50–150 ppm range and effectively evaluated the meat and sheep liver freshness in real-time [43]. Chang et al. [44] produced a detection system with an ultrasensitive amine gas sensor to perceive volatile amines in raw fish. Remarkably, the sensor offers an electrical response within 1 min that meticulously links total volatile basic nitrogen (TVBN) values. The sensor’s ppb-level sensitivity and integrated humidity control enable fast and accurate detection. These findings support the development of real-time, on-site freshness monitoring in fish processing environments. The amine gas sensor can detect ammonia, dimethylamine (DMA), and trimethylamine (TMA) at ppb levels, enabling it to monitor volatile compounds released from raw fish and indicate spoilage. The method truthfully releases the effects of storage temperature and a fish portion viz. ventral, dorsal, and lateral on spoilage development. For beltfish and mackerel, the sensors displayed a robust correlation with TVBN values.

Through in situ polymerization, Shi et al. [45] deposited TiO_2_-PANI into Silk Fibroin Fiber (SFF). The novel composite (TiO_2_-PANI/SFF) played the role of an excellent micro sensor exhibiting a sensing capability, with a response value equal to 0.82 and a response time of 10 s to 100 μg/L of NH_3_. In pork samples, the sensors used to evaluate freshness showed a strong correlation with TVB-N levels (R^2^ = 0.99). To measure TVBN more effectively, two non-destructive sensing methods, colorimetric sensors and hyperspectral imaging (HSI), were combined [46]. For data fusion and modeling, these authors proposed a BP-AdaBoost that corresponds to an effective backpropagation adaptive boosting algorithm. The performance of the model was examined relative to a PCA-BPANN: backpropagation artificial neural network model. The test results revealed that the data fusion model outdid the single-sensor models, with BP-AdaBoost proposing superior capability in handling complex data fusion compared to PCA-BPANN. In pork meat, this investigation revealed the possible integration of HIS and colorimetric sensors and the BP-AdaBoost algorithm for non-destructive TVB-N.

### 3.2. Biosensor-Based Detection of Microbial Hazards in Fish, Meat, and Meat Products

The occurrence of pathogenic microorganisms in food cause significant dangers to general health safety and can also affect the environment. Biosensor expansion has importantly improved food safety [47]. Conventional microbiological methods characteristically comprise enrichment, filtration, and incubation phases, requiring a time frame of 2–10 days to obtain [48]. Contrarily, modern biosensor-based tools proposed earlier have more precise detection, with the further advantage of on-site pertinence. For pathogens and their toxins and metabolites, their low detection limits the importance of highly sensitive analytical tools for guaranteeing fish, meat, and meat product safety.

Among different optical sensing methods, colorimetric, fluorescence, chemiluminescence SPR, and localized surface plasmon resonance (LSPR) are usually employed [49]. SPR-based biosensing normally engages reflectance spectroscopy for the detection of target pathogens, and the bioreceptors are fixed to a metal transducer surface. Specific wavelengths of electromagnetic radiation act towards the metal’s electrons and generate resonance. When bacterial cells attach to this surface, they induce quantifiable variations in the refractive index [47]. To detect pathogenic microorganisms in different meat and meat products, optical biosensors were employed. As an illustration, a fiber-optic immunosensor, fortified with immunomagnetic separation, certainly perceived *Listeria monocytogenes* in meat at levels as <3 × 10^2^ CFU/mL [50]. Another method employed an aptamer-based fiber-optic biosensor to select *L. monocytogenes* in artificially infected ready-to-eat (RTE) meat, effectively identifying it specifically among other microbial strains [51]. Oh et al. [52] engaged LSPR to detect *Salmonella Typhimurium* in pork at 4 log CFU/mL within 30 min. To synchronize the detection of *E. coli* O_157_:H_7_, *Salmonella enteritidis*, and *Listeria monocytogenes*, Zhang et al. [53] established an SPR biosensor combined with an enrichment broth. To simplify selective recognition, polyclonal antibodies that are special for each pathogen were anchored on separate channels of SPR chips. After an enrichment step, chicken meat was analyzed using the SPR system, efficaciously perceiving target microorganisms at 14, 6, and 28 CFU/25 g for *E. coli* O_157_:H_7_, *Salmonella enteritidis*, and *Listeria monocytogenes*, respectively. Liang et al. [54] produced a smartphone-based biosensor to detect microbial spoilage of ground beef. In this study, the lower limit of detection was between 10 and 100 CFU of *Escherichia coli* K_12_. Morant-Miñana and Elizalde [55] produced an electrochemical genosensor for *Campylobacter* spp. detection. This new material, developed from thin-film gold electrodes dropped onto Cyclo Olefin Polymer (COP), displayed high sensitivity, a robust linear response for *Campylobacter* spp., and positive authentication of real poultry meat samples. It displayed similar findings to those obtained with purified PCR products with a concentration range between 1 and 25 nM, and a LOD equal to 90 pM. Ohk and Bhunia [4] developed and optimized a multiplex fiber optic sensor able to simultaneously detect *L. monocytogenes*, *E. coli* O_157_:H_7_, and *S. enterica* in food samples. Streptavidin-coated optical sensors were equipped with biotinylated polyclonal antibodies and treated with bacterial suspensions or supplemented food samples for 2 h. In this study, turkey, ready-to-eat beef, and chicken samples were inoculated with ~10^2^ CFU of each pathogen/25 g and enriched for 18 h in a selective enrichment medium SEL broth and tested by the biosensor. The sensor positively recognized each pathogen individually or in combination, and the detection limit was 10^3^ CFU/mL for all three pathogens. This new approach, a multiplex fiber optic biosensor, could be appropriate for simultaneously detecting *Listeria*, *E. coli*, and *Salmonella* in food, decreasing the necessity for separate single-pathogen detection systems. By virtue of its excellent characteristics, like ultra-rapid electron transfer aptitude, great surface/volume ratio, suitability for biological applications, and its single connections with DNA bases of the aptamer, Muniandy et al. [56] fabricated an rGO-azophloxine nanocomposite (rGO-AP) aptasensor to detect foodborne pathogens. The contact of the label-free single-stranded deoxyribonucleic acid (ssDNA) aptamer with *S. Typhimurium* was examined by variance pulse voltammetry exploration, and this aptasensor indicated high selectivity and sensitivity for the detection of intact bacterial cells. rGO-AP revealed a linear detection range between 10 and 10^8^ CFU/mL and a good linearity (R^2^ = 98%). Furthermore, rGO-AP could detect bacterial concentrations ranging from 10 to 10^4^ CFU/g in chicken samples inoculated with *S. Typhimurium*. Rasooly [57] evaluated the potential of SPR biosensors to detect staphylococcal enterotoxin B (SEB), engaging 2 antibodies, in foods. A capturing antibody, covalently enclosed in the biosensor chip surface, performed the initial binding of the antigen, and a second antibody sticks to the captured antigen. Initially, the entire assessment cycle took 5 min when using a single antibody and 8 min when two antibodies were employed. Interestingly, the SPR biosensor could detect SEB in meat at 10 ng/mL, with initial binding at < 2 min. In another study conducted by Liu et al. [58], a fast detection of *Salmonella* serotypes B and D in ready-to-eat (RTE) turkey has been explored. These authors proved that the concentration of *Salmonella* <3 × 10^2^ cells/mL at 1 h was attained. Additionally, the findings displayed that the sensor can distinguish low concentrations of live *Salmonella* cells from high levels of dead *Salmonella* cells.

### 3.3. Biosensor-Based Detection of Contaminants, Antibiotics, and Drug Residues in Fish, Meat, and Meat Products

Food quality valuation includes perceiving impurities, such as drug residues, pesticides, toxins and heavy metals. Conventional tools, like mass spectrometry and capillary electrophoresis, are costly and require considerable time. To guarantee consumer security, biosensors offer a closer and gainful alternative with adequate perception. For instance, for heavy metals, such as Cd, As, and Hg, biosensors employed enzymes (e.g., GOx, urease, cholinesterase, alkaline phosphatase) and genetically modified microorganisms [59]. By developing a chemiluminescence sensor called MIP (molecularly imprinted polymer (MIP), Cai et al. [60] recognized eight benzimidazoles in beef and mutton, establishing ultrafast sensitivity. In fact, these authors confirmed that the detection limits ranged between 1.5 and 21 pg/mL, with 18 min, and had a high recovery efficiency (66–91%). To identify fungal or bacterial toxins existing in meat products, electrochemical biosensors are used. As an example, trichothecene (T-2 toxin) was detected in swine meat [61]. By employing an electrochemical and SPR biosensors, Staphylococcal enterotoxin B was sensed in pork [62] and potted meat [57], respectively. Using an amperometric biosensor, Dinçkaya et al. [2] appraised the nitrate concentrations in meat and confirmed that the LOD was 2.2 × 10^−9^ M with a response time equal to 10 s. On the other hand, some studies employed SPR as biosensors to identify drug residues. In several meat species like pork, beef, and chicken, SPR technique was able to detect sulphonamides and chloramphenicol have been quantified [62,63,64,65]. In order to detect the SDM: sulfadimethoxine in beef and chicken meat, Mohammad-Razdari et al. [66] established an electrochemical biosensor based on a pencil graphite electrode (PGE) and adapted with a reduced graphene oxide (RGO) and Au nanoparticles for sulfadimethoxine (SDM). In the best-performing trials, the proposed biosensor showed a linear range from 10 to 10^−5^ M and a LOD at 3.7 × 10^−16^ M towards SDM. For meat sample applications, the aptasensor was applied to fish, chicken, and beef and showed acceptable recovery rates across the tested concentration range, demonstrating dependable performance and accuracy in analytic quantification between 92 and 103%. For the label-free detection of ceftiofur residues in meat trials, Stevenson et al. [67] developed an affinity-based electrochemical biosensor. These authors validated a platform that could detect ceftiofur within 15 min of using the sample at levels down to 0.01 ng/mL in phosphate-buffered saline and 10 ng/mL in 220 mg ground turkey meat samples. Table 2 summarizes some examples of biosensors for monitoring the quality and safety of fish, meat, and meat products.

## 4. Standardization and Validation of Biosensors in Real-Time Food Quality Monitoring

The standardization and validation of biosensors are indispensable processes for ensuring their reliability, reproducibility, and regulatory acceptance in the food industry. Unlike conventional chemical assays, biosensors often demonstrate significant variability due to differences in biological recognition elements, sensor fabrication, and susceptibility to environmental factors, such as temperature, pH, and matrix complexity. This variability makes robust calibration and method validation protocols necessary for ensuring consistent performance across food matrices and operational environments [81,82].

Calibration and standardization are foundational steps for establishing accuracy and consistency in biosensor output. The calibration process typically involves the use of matrix-matched reference standards, ideally certified, to reflect real-world food conditions in terms of composition, viscosity, and potential interferents [83]. Biosensors must exhibit predictable and linear responses across a defined concentration range of the target analyte. For example, a heat-transfer biosensor used for detecting trace levels of chemical additives in dairy was calibrated using milk samples with varying fat contents to ensure consistent sensitivity and reproducibility [10].

### 4.1. Validation

**Figure 5:**
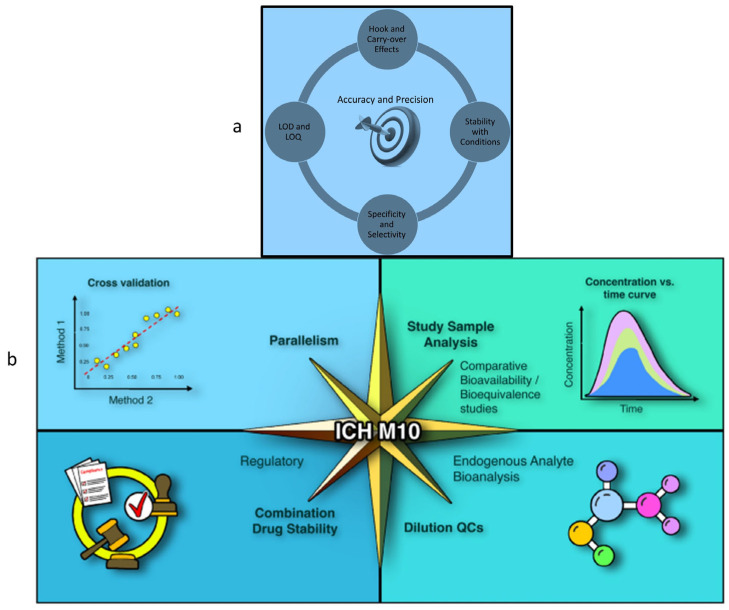
Bioanalytical method validation. (**a**) Key biosensor validation parameters. (**b**) Various aspects of bioanalytical method validation (reproduced from Vazvaei-Smith et al. [88] with permission from the AAPS Journal, copyright 2024).

Although harmonized guidelines for validation of biosensor-based methods do not exist, a valid text is represented by the International Council for Harmonisation (ICH) “Bioanalytical Method Validation and Study Sample Analysis—M10 guideline, 2022” [84]. This document was adopted also by the European Medicines Agency (EMA) and the Food and Drug Administration (FDA). Method validation, as defined in the ICH M10 guideline, requires comprehensive evaluation of analytical performance. Key parameters include accuracy, precision (repeatability and intermediate precision), selectivity, sensitivity, linearity, LOD, limit of quantitation (LOQ), carryover, and analyte stability [85,86,87,88]. The main concepts in bioanalytical method validation and key biosensor validation parameters are illustrated in Figure 5.

#### 4.1.1. Specificity and Cross-Reactivity Challenges

In biosensor-based ligand binding assays (LBA), specificity refers to the sensor’s ability to detect only the target analyte without interference from structurally similar compounds, such as analogues, metabolites, or co-formulated substances. This becomes critical when detecting contaminants, like veterinary drug residues or pesticide metabolites. Specificity is typically evaluated by spiking blank matrix samples with structurally related compounds at their expected maximal concentrations. A well-validated biosensor should show negligible response to these analogues and maintain accuracy for the primary analyte within ±25% at the extremes of its dynamic range. In cases in which specificity is compromised, adjusting the quantification range or employing alternative recognition elements (e.g., more selective antibodies or aptamers) may be necessary.

#### 4.1.2. Selectivity in Complex Food Matrices

Selectivity addresses the biosensor’s performance in distinguishing the analyte from endogenous matrix components that may interfere with detection. This is especially challenging in samples, such as milk, eggs, or processed foods, where proteins, fats, and enzymes can cause non-specific binding or signal suppression. To ensure selectivity, the assay must be tested in at least 10 different blank food matrix samples, with analytes spiked at both low and high concentrations. The signal from unspiked samples should fall below the lower LLOQ in at least 80% of the matrices tested. Selectivity testing should also consider lipemic and hemolyzed conditions, as well as matrices derived from diseased or stressed animal populations when relevant.

#### 4.1.3. Calibration Curve and Reportable Range

Accurate quantification with biosensors depends on the establishment of a calibration curve, relating the analyte concentration to the signal response. The curve should span from the LLOQ to the upper limit of quantification (ULOQ), ideally covering at least six concentration points plus a blank. Many biosensor platforms use a logistic fit (4- or 5-parameter models) to accommodate non-linear signal responses, especially near saturation zones. A robust calibration curve requires consistency across multiple runs (minimum of six), with at least 75% of calibration points meeting accuracy criteria (±25% at LLOQ/ULOQ; ±20% at other levels).

#### 4.1.4. Accuracy and Precision Requirements

Validation of accuracy (closeness to the true value) and precision (repeatability) is conducted using quality control (QC) samples at multiple concentration levels, typically LLOQ, low, medium, high, and ULOQ. Within-run and between-run performance should be assessed over at least six analytical runs using independently prepared QCs. Acceptable accuracy and precision limits are ±20% (±25% for LLOQ and ULOQ). A total error (sum of bias and variability) threshold of ≤30% (≤40% at extremes) is often applied as an overall acceptance criterion.

#### 4.1.5. Dilution Linearity and High-Dose Hook Effect

Due to the limited dynamic range of many biosensors, the dilution of samples with high analyte concentrations is necessary. Dilution linearity must be verified to ensure that sample dilution does not introduce bias. This is also critical for identifying the hook effect, a phenomenon in which excessive analyte concentrations saturate binding sites, leading to signal suppression. Dilution series should be tested in at least three independent preparations, demonstrating linearity across the measured range, with ≤20% deviation from expected values.

#### 4.1.6. Stability Under Analytical Conditions

Stability testing ensures that storage, processing, and handling conditions do not compromise the biosensor’s performance. This includes assessments of freeze–thaw stability, bench-top stability, and long-term storage. For each condition, QCs at low and high concentrations should be evaluated, and analyte recovery should remain within ±20% of nominal values. This step is particularly important for biosensors using biologically active components (e.g., enzymes or antibodies), which are prone to degradation under suboptimal storage.

These criteria ensure the biosensor ability to generate reliable results for target contaminants, such as pesticides, preservatives, or industrial pollutants [85]. These validation criteria should be tailored depending on whether the biosensor detects contaminants, chemical additives, toxins, or other analytes. A critical point is also food matrices; in fact, the ICH M10 Guideline underlines that other pivotal parameters are matrix effects, incurred sample reanalysis (ISR), and inter-batch reproducibility, all of which are particularly relevant for biosensors deployed in complex food matrices, like oils, processed meats, and lipid-rich seafood (e.g., shellfish) [89,90].

While the ICH M10 guideline does not explicitly refer to “measurement uncertainty” in metrological terms, it does encompass key contributors to uncertainty through required validation parameters, such as accuracy, precision, LOD, LOQ, and total error. These collectively influence the uncertainty of biosensor measurements. In the context of biosensors, where environmental and matrix effects can further amplify variability, it may be valuable for future regulatory guidance or standardization efforts to integrate formal uncertainty estimation.

In Table 3, a summary of these parameters as well as a brief description is reported.

Despite innovative sensor designs, regulatory approval remains a time-intensive process. In fact, often, apart from a validation study, a comparison study with validated chemical reference methods, such as HPLC or mass spectrometry, is preferred. These comparative assessments are crucial for establishing biosensor equivalence in terms of sensitivity, selectivity, and reproducibility. Without this level of validation, biosensors face challenges in gaining acceptance for routine food safety monitoring, despite offering advantages, such as portability and real-time readouts [91,92].

Biosensor integration into quality control systems presents operational challenges, including interoperability with digital traceability platforms, training personnel in sensor operation, and upgrading existing laboratories or processing infrastructure. In large-scale manufacturing environments, biosensor data must seamlessly interface with automated decision-support systems for tasks, such as batch release or contamination alerts [93,94,95].

Furthermore, data harmonization is critical. Standardized biosensor outputs must be structured and formatted for compatibility with central databases that consolidate information from inspections, internal audits, and supply chain feedback. As highlighted by Wijayanti et al. [96], biosensors are increasingly incorporated into the digitalization of food quality frameworks, but effective deployment requires unified validation standards and interoperable data formats to enable real-time risk assessment and traceability [97].

### 4.2. Limits and Challenges for Biosensor Application in Real-Time Food Quality Monitoring

Achieving high sensitivity and specificity remains a central challenge in the development of biosensors for detecting food additives and contaminants. These parameters determine the biosensor’s ability to detect target analytes at trace levels and to discriminate them from structurally similar compounds. In complex food matrices, such as milk or cereals, matrix components can interact with sensor surfaces or recognition elements, leading to background signal noise or false positives [97].

For instance, certain immunoassays for mycotoxins have demonstrated cross-reactivity with masked or metabolized toxin forms, undermining their selectivity. Similarly, surface-enhanced Raman scattering (SERS)-based lateral flow biosensors developed for detecting colistin in milk have shown matrix interference from milk proteins, which reduced analytical clarity despite fast detection times. Such cases highlight the need for advanced recognition elements and sample pre-treatment strategies to mitigate matrix effects and improve signal fidelity. Moreover, the operational stability of biosensors, especially those incorporating biological recognition elements, like enzymes or antibodies, is a persistent issue limiting their shelf-life. Enzyme-based biosensors are particularly susceptible to denaturation or leaching during storage, which reduces signal reproducibility and overall reliability [98,99].

Efforts to improve stability have focused on immobilization techniques, such as cross-linking, encapsulation in polymeric matrices, or covalent bonding to support materials. These approaches aim to preserve the functional conformation of the biomolecules and enhance resilience to environmental stressors during storage and use. However, long-term validation of such methods under varied food storage conditions remains limited and is critical for regulatory and industrial acceptance. Another critical issue may be represented by environmental factors, including temperature, humidity, and pH, which have a significant impact on biosensor performance. Temperature fluctuations can alter enzyme kinetics, signal generation rates, or the refractive index in optical systems. For example, enzymatic biosensors may show exaggerated signals at elevated temperatures or delayed responses in colder environments. Similarly, pH instability affects the electrochemical response of sensors, especially those incorporating carbon nanomaterials for detecting heavy metals or preservatives [100].

Humidity can degrade sensitive components, particularly in optical biosensors, in which uncontrolled moisture introduces signal noise or damages light-sensitive dyes. Moreover, food matrices with variable composition further complicate biosensor operation, reinforcing the need for robust calibration and compensation mechanisms to ensure consistent performance [101].

Some examples of issues in biosensor validation and application for the analysis of food additives and contaminants, along with the study strategies developed for their resolution, are proposed in Table 4.

## 5. Challenges, Limitations, and Future Perspectives in Biosensor Applications for Fish, Meat, Poultry, and Related Product Safety Monitoring

Addressing the challenges and limitations in ensuring the safety of fish, meat, poultry, and related products is a fundamental pillar of modern food systems. However, biosensors—despite their transformative potential—still face multifaceted limitations that restrict their scalability and real-world implementation. These challenges span across biological, technical, regulatory, and economic domains, especially in resource-limited settings or small-to-medium-scale enterprises.

One of the most fundamental limitations stems from the complexity of food matrices, which vary widely in moisture content, fat and protein composition, and microbial load. These intrinsic properties can interfere with biosensor readings, especially in systems relying on electrochemical or optical signals [97,106,107,108]. High-fat samples, like beef or lamb, may cause signal drift or fouling of the sensing surface, while the high-water activity in fish products may lead to enzymatic degradation or dilution of target analytes [97,106]. Additionally, meat and poultry tissues can contain a mixture of endogenous enzymes and oxidation byproducts that further complicate signal stability [107,108]. In seafood, detection is further complicated by the presence of marine-specific hazards, such as tetrodotoxin, okadaic acid, or domoic acid, which require ultra-sensitive detection limits and matrix-adapted recognition elements [106,109,110]. In poultry, early stage detection of infection is difficult due to low biomarker concentrations during the asymptomatic phases of disease progression, which often fall below the LOD of many conventional biosensors [107,111].

Cross-reactivity and specificity pose another technical barrier. Biosensors must be able to differentiate between highly similar microbial species or strains, such as *Campylobacter jejuni* versus *C. coli*, or between pathogenic and non-pathogenic *E. coli* strains, which often share structural markers [97,112,113]. The inability of many biosensors to discriminate between viable and non-viable cells may lead to false positives, especially in post-sanitization environments [112,114]. Moreover, many detection platforms still struggle with achieving the necessary selectivity in mixed microbial environments, particularly in raw or minimally processed products [106,115,116].

Furthermore, detection of residues, such as tetracyclines or aflatoxins—particularly in trace amounts across different feed types, tissues, or products—requires extremely sensitive and consistently calibrated platforms [116,117,118,119,120,121]. Small deviations in temperature, pH, or sample handling can cause shifts in biosensor response, making reproducibility a serious concern for both researchers and industry practitioners [97,100,122].

Operational challenges are also significant. Electrochemical biosensors often require external power sources and supporting instruments (e.g., potentiostats), which hinder their portability and real-time usability in field inspections [123,124,125,126]. Similarly, colorimetric biosensors, despite their visual simplicity, tend to require multi-step sample preparation and are vulnerable to variations in ambient lighting or subjective interpretation, especially in environments lacking standardized conditions [127,128]. Smartphone-based visual readers are being tested to mitigate these issues, but their precision and user-friendliness still vary widely [129,130]. In contrast, SERS-based biosensors, while capable of ultra-sensitive detection, are technically demanding due to their reliance on precision optical components (e.g., Raman lasers, detectors) and the need for specialized substrates such as gold or MOF-coated nanoparticles [110,130,131,132,133,134,135]. Additionally, there is no universal SERS substrate that can accommodate all analyte types, necessitating tailored fabrication for each application [131,135,136].

Moreover, optical and SPR technologies provide label-free, real-time monitoring capabilities that are especially suited to packaging and food processing environments [137,138], but they require precise optical setups, which limit portability.

From a regulatory and commercial perspective, biosensor platforms are not yet widely incorporated into formal food safety systems, such as HACCP, ISO 22000, or Codex Alimentarius frameworks. Validation against gold-standard methods (e.g., culture-based enumeration, ELISA, or qPCR) is still lacking for many sensor formats, which affects their credibility in audits, certifications, and trade compliance [122,139,140,141]. The absence of harmonized validation protocols makes it difficult to compare results across borders or industries, leading to skepticism among food producers and regulatory bodies alike [97,118].

On the economic front, biosensors incorporating nanomaterials, CRISPR technology, or microfluidics often have high development and production costs, especially when coupled with surface functionalization and antibody/aptamer design [139,142,143,144,145,146]. This restricts their use in lower-income regions or small-scale food businesses. Scaling up from laboratory prototypes to commercial-grade devices often requires substantial investment in cleanroom facilities, testing, and certification [147]. Moreover, concerns regarding the long-term environmental and human health impacts of nanomaterials (e.g., silver nanoparticles, graphene oxide) continue to raise regulatory red flags, necessitating the shift toward green synthesis and biodegradable materials [148,149,150]. The need for non-toxic, disposable sensor platforms is gaining traction in global sustainability goals [148].

Biosensors also have an emerging role in monitoring cultured (cell-based) meat, a sector with specific challenges related to contamination control during cell cultivation, the composition of growth media, and the use of biochemical additives [151,152].

Finally, while point-of-care (POC) and intelligent packaging biosensors are increasingly being designed, real-world uptake is still slow. High costs, difficulty in integrating sensor data into existing software ecosystems, and energy requirements for continuous operation (especially for cold chains or remote sites) further hinder long-term monitoring applications [153,154]. In addition, data security and interoperability challenges persist, particularly when transferring biosensor data to cloud-based regulatory or logistics systems [140,141].

Despite the broad range of challenges, biosensor development is progressing rapidly, supported by innovations in nanotechnology, synthetic biology, electronics, and digital infrastructure. The next generation of biosensors is being engineered to meet not only technical performance benchmarks but also criteria for usability, affordability, and sustainability.

A key design philosophy is alignment with the REASSURED framework—real-time connectivity, ease of sample collection, affordable, sensitive, specific, user-friendly, rapid, robust, equipment free, and deliverable to end users [5,6]. Recent advancements in lab-on-a-chip (LOC) systems and wearable biosensors now allow continuous monitoring of animal stress biomarkers (e.g., cortisol, IL-6), meat spoilage indicators (e.g., biogenic amines), or microbial contamination in real-world environments [155,156,157,158]. LOC devices can be used directly in slaughterhouses, packaging lines, or distribution centers, reducing delays between contamination and detection.

Integration of biosensor data with IoT platforms, blockchain-enabled traceability, and AI-driven analytics is revolutionizing food safety by enabling predictive diagnostics and real-time response. For example, blockchain can secure biosensor data logs for traceable certification, while AI algorithms can analyze spectral or electrical patterns to detect anomalies or mixed contaminations [159,160,161,162,163,164]. AI-based decision-support systems can also be trained in biosensor outputs to guide preventive measures in processing plants or farms. Smartphone-enabled biosensors also bridge accessibility gaps by allowing frontline inspectors and small producers to capture and transmit results immediately, often with GPS and timestamp metadata [164]. These solutions support decentralized decision-making and democratize food safety monitoring [165,166].

The frontiers of biosensor technology are also being expanded through multiplexing and advanced signal amplification. CRISPR-Cas systems offer unparalleled specificity at attomolar levels, enabling detection of pathogens, like *Listeria monocytogenes*, *E. coli* O157:H7, or *Salmonella enterica*, in complex matrices [167,168]. Meanwhile, nanozyme-based colorimetric sensors provide robust alternatives to enzyme-based assays, maintaining stability under diverse environmental conditions and simplifying fabrication [169,170,171,172]. Nanozymes also eliminate cold-chain dependence for sensor reagents [171].

Multi-analyte aptasensors are being designed to simultaneously detect microbial pathogens, spoilage indicators, and chemical toxins in a single run—dramatically improving throughput and cost-effectiveness [104,115,173]. Similarly, molecularly imprinted polymer (MIP)-based sensors show high selectivity for volatile markers, like histamine or trimethylamine, offering practical applications for seafood spoilage detection [174,175]. Such developments are particularly useful in import–exports, where spoilage needs to be evaluated rapidly at ports or distribution hubs.

In the realm of packaging, intelligent sensors are now integrated directly into films, labels, or coatings to detect changes in gas composition (e.g., CO_2_, NH_3_), humidity, or microbial growth. These include Hx-sensing films for fish freshness and polymyxin B-aptamer platforms for endotoxin detection in poultry products [176,177,178,179,180]. Emerging solid-state SERS substrates (e.g., paper, elastomers, AuNS-glass composites) also offer durability and reusability in smart packaging applications [181,182,183,184]. Such features make them attractive for both consumers and regulatory audits.

To overcome energy and maintenance concerns, self-powered biosensors using biofuel cells or photoelectrochemical modules are being tested for autonomous deployment in storage environments with minimal infrastructure [154]. These devices align well with sustainability goals and reduce the carbon footprint of food monitoring.

Ultimately, the successful integration of biosensor technologies into food safety systems will rely not only on overcoming technical and operational barriers but also on establishing robust regulatory frameworks and fostering international standardization. It is essential that biosensor data be recognized as legally valid and interoperable across digital platforms used in global supply chains. Interdisciplinary collaboration among scientists, technologists, policymakers, and industry stakeholders will be key to accelerating the transition from research prototypes to field-deployable, validated tools.

By addressing current limitations in sensitivity, matrix interference, cost-effectiveness, and data integration, biosensors can be positioned as core components of intelligent, sustainable, and resilient food safety systems for the fish, meat, and poultry industries.

## 6. Conclusions

Biosensors have emerged as transformative tools for ensuring the safety and quality of fish, meat, poultry, and related food products. Their capacity to rapidly and sensitively detect contaminants, pathogens, spoilage markers, and drug residues positions them as viable and often superior alternatives to conventional laboratory-based methods. Recent advancements including the integration of nanomaterials, lab-on-a-chip platforms, smartphone interfaces, and IoT connectivity have significantly enhanced their portability, usability, and real-time monitoring capabilities.

Nevertheless, several critical challenges remain. The complexity of food matrices, environmental variability (such as pH, humidity, and temperature), and the inherent instability of biological recognition elements can affect performance and limit reproducibility. Regulatory acceptance is further constrained by the absence of harmonized validation standards and insufficient comparative assessments with gold-standard analytical techniques. Additionally, high development and implementation costs hinder widespread adoption, particularly in resource-limited settings.

Looking forward, the development of biosensors should prioritize robustness, affordability, and compliance with international regulatory frameworks. Embracing the REASSURED criteria, ensuring that devices are real-time, easy to use, affordable, sensitive, specific, user-friendly, rapid, robust, equipment free, and deliverable to end users, will be key to broader deployment. Integration with AI-powered analytics and blockchain-based traceability systems can also unlock new opportunities for predictive diagnostics and transparent supply chain management. With continued interdisciplinary collaboration and innovation, biosensors are well-positioned to become cornerstone technologies in next-generation food safety and quality assurance systems.

## Figures and Tables

**Table 1:** Type of biosensor employed for fish, meat, poultry, and related product quality and safety monitoring.

Target Analyte	Food Product	Bioreceptor	Transducer/Electrode	Data Visualization Device	Limit of Detection (LOD)	Sensitivity/Linear Range/Detection Time	Reference
Histamine	Fish	DAO or monoamine oxidase (MAO) enzymes	Electrochemical Screen-printed carbon electrodes	Cyclic voltammetry (CV), chronoamperometry, and EIS	-	Sensitivity 8.957 × 10^−2^ mM	[19]
Putrescine	Beef, pork, chicken, turkey, and fish meat	Putrescine oxidase	Electrochemical	Chemiluminescence Microplate luminometer		linear range 0.8–2 mg/L	[15]
Xanthine	Chicken meat	Guanine deaminase and XOD	Electrochemical with fiber optic probe	Spectrometer (OceanOptics)	-	Sensitivity 44 µM at 5 days	[16]
Nitrate	Meat sample	Nitrate reductase	Ag/AgCl reference electrode, platinum auxiliary electrode, and working electrode glassy carbon (GCE)	Voltametric analysis	2.2 × 10^–9^ M		[2]
Glucose reduction	Beef meat	GOx	Glassy carbon electrode modified with multi-walled carbon nanotubes and chitosan	CV, differential pulse voltammetry, and EIS	-	linear range 0.01–0.06 mmol/L	[17]
Chloramphenicol (CAP)	Beef and pork meat samples	Monoclonal antibody to CAP (anti-CAP)	Electrochemical immunosensor and ESI techniques	ESI	0.06 ng/mL	-	[3]
Calpastatin	Beef meat	Primary anti-calpastain antibody and secondary enzyme-labelled antibody	Potentiostat–galvanostat with Gold working (W.E.) and counter (C.E.) electrodes silver pseudo-reference electrode	Amperometric detection	-	Sensitivity 481 ng/mL	[20]
Tetracycline	Poultry muscle samples	Lyophilized reconstituted sensor cells	Cell-biosensor	Bioluminescence with SynergyTM HT Multi-detection Microplate Reader		Sensitivity: 10 µg/kg	[21]
DNA (Donkey meat)	Donkey adulteration in cooked sausages	DNA	Multi-parameter SPR device with gold chips	SPR	1.0 nM	-	[5]
Dopamine Adulterant	Beef meat	Anti-dopamine substance	-	Colorimetric sensor	0.13 mM	-	[22]

**Table 2:** Biosensors for monitoring the quality and safety of fish, meat, and meat products.

Target Analyte	Bioreceptor	Immobilization Technique	Food Product	LOD	Sensitivity/Linear Range/Detection Time	References
Some examples for biosensors that detect freshness in fish, meat, and meat products.						
Hx	XOD within a Nafion matrix on a graphene–titanium dioxide	Entrapment	Pork	9.5 μM	Sensitivity: 4.1 nA/μM Linear range 20–512 μM	[6]
	XOD and horseradish peroxidase	Adsorption	Raw and treated meat samples	1.8 mg/L	Quantitative limit: 6.1 mg L^−1^	[34]
	XOD and polyvinylferrocenium perchlorate matrix on a platinum	Adsorption	Fish	0.6 μM,	Linear range 2.1–103 μM	[68]
	XOD and platinum electrode with single-walled carbon nanohorns (SWCsNH) and gold nanoparticles (AuNP)	Covalent	Fish	0.61	Linear range 1.5–35.4	[69]
	XOD and uricase within a polypyrrole-paratoluenesulfonate composite film	Entrapment	Fish	5	5–500 Linear range	[70]
	XOD on carbon film electrodes and carbon nanotube	Cross-linking	Fish	0.77	10–130	[71]
	XOD onto a modified platinum electrode surface	Entrapment	Seafood	0.0023	0.01–10	[70]
	XOD onto paper substrate	Adsorption	Fish	4.1	4–35	[72]
Calpastatin	Capillary and optical fiber biosensor	Covalent	Longissimus muscle from beef		Calpastatin activity (R^2^ = 0.6058)	[73]
Cadaverine	Receptor molecules onto the surface of thiol-gold	Covalent	Beef, chicken, or pork			[74]
Putrescine	Casein onto the electrode surface using glutaraldehyde	Covalent	Beef, pork, chicken, turkey meat samples	0.8 mg/L–1.3 mg/L	Linearity range: 1–2 mg/L	[75]
TVBN	pre-fabricated responsive dyes, embedded onto a paper or polymer film	Adsorption	Pork meat		Correlation coefficient (R^2^ = 0.932)	[46]
Some examples of biosensors for detecting pathogenic microorganisms and toxins in meat and meat products.						
*Campylobacter* spp.	Amino-modified DNA probes onto a nylon membrane	Covalent	Chicken meat	3 pg/μL of DNA	-	[28]
*Salmonella enterica*, *Listeria monocytogenes,* and *Escherichia coli* O157:H7	antibodies onto the optical fiber surface using carbodiimide	Covalent	Beef, turkey breast and chicken	10^3^ CFU/mL	-	[4]
*Salmonella Typhimurium* *Staphyloccocus aureus*	Thiol-modified aptamers onto gold nanoparticles	Non-covalent	Pork	15 CFU/mL 35 CFU/mL	Recovery rate: 94.12–108.33%	[53]
*S. enterica* serovar *Typhimurium*	Amine-terminated DNA aptamers onto a carboxyl-functionalized graphene-modified electrode employing carbodiimide	Covalent	Chicken meat	1 CFU/mL	Linear range (detection): 1–8 log CFU/mL	[56]
*Salmonella pullorum*	specific antibodies onto the electrode surface using glutaraldehyde	Covalent	Chicken meat	100 CFU/mL	Detection time: 1.5 to 2 h	[76]
*E coli* K-12	specific antibodies onto the gold electrode surface	Adsorption	Chicken meat	3 log CFU/mL	-	[77]
*Listeria monocytogenes*	thiol-modified DNA aptamers onto gold nanoparticles	Covalent	Meat samples	2 log CFU/g	Linear detection range: From 10^2^ to 10^7^ CFU/m Detection time <30 min	[51]
*L. monocytogenes* toxin *S. aureus* enterotoxin B	Live mammalian cells onto the surface of gold interdigitated microelectrodes	Adsorption	Salami	10^4^ CFU/mL 100 ng/mL	Detection time < 1 h	[78]
Staphylococcal enterotoxin B	Anti-SEB antibodies onto a gold-coated SPR	Covalent	Meat	0.5 ng/mL	0.5 ng/mL to 20 ng/mL Detection time <20 min	[57]
Trichothecene T-2 toxin	Anti-T-2 toxin antibodies onto a modified electrode surface using glutaraldehyde	Covalent	Swine meat	0.04 ng/mL	0.05–20 ng/mL Detection time = 30 min	[61]
Some examples for biosensors detecting antibiotics, drug residues, and additives in meat products						
Tetracyclines	*E. coli* cells in agarose gel on the surface of microplates or membrane	Entrapment	Poultry muscle samples	2–5 µg/kg	2 to 100 µg/kg Detection time = 3 h	[26]
CAP	CAP–protein conjugate onto the SPR sensor chip	Covalent	Poultry muscle	100 ng/kg	0.1 to 1 µg/kg detection time <30 min	[79]
Oxytetracycline (OTC) Kanamycin (KAN) Ampicillin (AMP)	Aptamers onto citrate-stabilized gold nanoparticles	Adsorption	Chicken	0.42 ng/mL 0.31 ng/mL 0.2 8 ng/mL	1–100 ng/mL 1–80 ng/mL 1–60 ng/mL Detection time = 15 min	[80]
Ractopamine	Ractopamine–BSA conjugate onto a carboxymethylated dextran chip	Covalent	Pork	0.09 ng/mL	0.1–10 ng/mL Detection time = 10 min	[81]

**Table 3:** Summary of key validation parameters for biosensor-based methods according to ICH M10 guidelines.

Parameter	Definition	Regulatory Expectation	Biosensor-Specific Considerations
Specificity	Ability to detect only the target analyte, not structurally similar compounds	Interference from related compounds should result in <LLOQ signal; accuracy ±25% at extremes	Biosensors using antibodies/aptamers must be screened against analogs, metabolites, and additives
Selectivity	Differentiation of analytes in the presence of matrix components	≥80% of blank matrices should show <LLOQ signal; accuracy within ±25% at LLOQ	Must account for interference from fats, enzymes, or proteins common in food matrices
LOD	Lowest concentration distinguishable from blank with confidence	typically signal/noise (S/N) ≥3	Important for contaminant detection; impacted by sensor noise and baseline stability
LOQ	Lowest concentration quantifiable with acceptable accuracy and precision	S/N typically ≥10	Defines lower end of calibration; matrix effects often limit LOQ in real food samples
Calibration Curve	Relationship between analyte concentration and sensor response	≥6 levels + blank; logistic fit often used; 75% points within ±20–25% of nominal value	Non-linear response at low/high ranges often requires 4-/5-parameter modeling
Accuracy	Closeness of measured value to true value	Within ±20% (±25% at LLOQ/ULOQ); evaluated within- and between-runs	Challenging when sensor drift or matrix effects occur; needs robust QC planning
Precision	Repeatability of results under same conditions	CV ≤20% (≤25% at LLOQ/ULOQ); across ≥6 runs and 5 QC levels	Signal variability from biorecognition elements (e.g., enzyme-based biosensors) must be managed
Total Error	Sum of bias (accuracy) and variability (precision)	Should not exceed 30% (40% at LLOQ/ULOQ)	A helpful global indicator of biosensor method performance
Dilution Linearity	Consistency of measurement across diluted samples	Mean ±20% of expected after correction; ≥3 dilutions tested	Needed for samples exceeding range; verifies absence of hook effect
Hook Effect	Signal suppression at high analyte concentrations	No signal drop-off in undiluted samples expected above ULOQ	Particularly relevant in immunoassay-based biosensors
Carry-over	Residual analyte signal from prior sample influencing subsequent results	Signal in blank after ULOQ standard must be <LLOQ	Typically minimal in biosensors; confirm with blank after high calibrator
Stability	Analyte remains unchanged during storage, preparation, and analysis	Mean ±20% at low/high QC; validated over actual storage conditions	Biosensor reagents (e.g., enzymes, aptamers) and analyte stability must both be validated

**Table 4:** Biosensor applications for food additives and contaminants: key challenges and mitigation strategies.

Analyte	Biosensor Type	Matrix	LOD/LOQ	Key Challenges	Mitigation Strategies	References
Carbendazim	upconversion-MnO_2_ luminescent resonance energy transfer	food	0.05 ng·mL^−1^	specificity	aptamer integration and high fluorescence quenching capability of MnO_2_ nanosheets	[101]
cadmium (Cd), lead (Pb) and mercury (Hg)	luciferase-based biosensors	food	Cd: 0.01 μM Pb: 0.025 nM Hg: 2 nM	decrease of sensitivity	expression of Pb importers or nonspecific modifications	[102]
Nitrate	Immobilized Nitrate Reductase	dry-cured ham	-	comparison with HPLC	good agreement with standard HPLC method: R^2^ = 0.971	[103]
amnesic shellfish toxins: domoic acid	Aptamer-Based Biosensor	-	13.7 nM	specificity	identification and truncation optimization	[104]
Paralytic Shellfish Poisoning Toxins	Surface Plasmon Resonance-Based Biosensors	shellfish	-	interferences	comparison of several extraction methods	[105]

## Data Availability

No new data were created or analyzed in this study. Data sharing is not applicable to this article.
